# Characteristic Tearing Energy and Fatigue Crack Propagation of Filled Natural Rubber

**DOI:** 10.3390/polym13223891

**Published:** 2021-11-10

**Authors:** Jigang Rong, Jun Yang, Youjian Huang, Wenbo Luo, Xiaoling Hu

**Affiliations:** 1 School of Packaging and Materials Engineering, Hunan University of Technology, Xiangtan 411105, China; jihangrong@gmail.com; 2 Zhuzhou Times New Material Technology Co., Ltd., Zhuzhou 412000, China; 201731570117@smail.xtu.edu.cn; 3 College of Civil Engineering and Mechanics, Xiangtan University, Xiangtan 411105, China; luowenbo@xtu.edu.cn

**Keywords:** filled natural rubber, characteristic tearing energy, cutting experiment, fatigue crack propagation

## Abstract

Below the incipient characteristic tearing energy (*T*_0_), cracks will not grow in rubber under fatigue loading. Hence, determination of the characteristic tearing energy *T*_0_ is very important in the rubber industry. A rubber cutting experiment was conducted to determine the *T*_0_, using the cutting method proposed originally by Lake and Yeoh. Then, a fatigue crack propagation experiment on a edge-notched pure shear specimen under variable amplitude loading was studied. A method to obtain the crack propagation rate d*a*/d*N* from the relationship of the crack propagation length (Δ*a*) with the number of cycles (*N*) is proposed. Finally, the *T*_0_ obtained from the cutting method is compared with the value decided by the fatigue crack propagation experiment. The values of *T*_0_ obtained from the two different methods are a little different.

## 1. Introduction

Rubber is widely used in industrial field due to its excellent mechanical properties. In the traditional fields, rubber is used for tires, driving belts, seismic isolators, engine bushing and so on [1,2]. Nowadays, it is emerging in new fields, such as soft robotics, stretchable electronics, tissue engineering and others [3,4,5]. In these applications, soft rubbers often suffer complex cyclic loads. When suffering cyclic loads, catastrophic failure frequently induced in rubbers caused by crack propagation. During the fatigue crack propagation process, the incipient tearing energy (*T*_0_) is very important for the anti-fatigue property of rubber [6]. Below *T*_0_, cracks will not grow in rubber under fatigue loading. *T*_0_ is generally regarded as a material constant of fatigue resistance. To prevent the fatigue failure, one strategy is to improve the *T*_0_ of rubber. Thus, measuring the *T*_0_ has caught much attention.

There are various approaches for quantifying *T*_0_. One is near-threshold fatigue crack propagation measurements. This method can get the *T*_0_ easily, but it requires a long duration of continuous testing [7]. Lake and Thomas [8] proposed a model to calculate the incipient characteristic tearing energy of an elastomer on an arbitrary orientation plane. Although the theoretical and experimental results are in good agreement, the model is complex and difficult to calculate. A cutting method which is easy and can obtain the *T*_0_ in a short time was proposed by Lake and Yeoh [9]. Chang and Wang [10] suggested that cutting tests are capable of conveniently measuring the fracture toughness of polymers; however, the results are affected by the cut depth and the sharpness of cutting tools. Zhang et al. [11] investigated the effects of the cutting tool and cutting rate on the tearing energy using a “Y-shaped” rubber sample. Due to the convenience of the cutting method, the classic Lake-Yeoh cutting method was even introduced into an Intrinsic Strength Analyser (ISA), which is a commercial testing instrument and can test the *T*_0_ in two hours [7]. Moreover, new methods were proposed to decide the characteristic tearing energy of a rubber material by analyzing the profile of the crack surface or the crack tip shape during the tearing process [12,13]. Since the characteristic tearing energies are very important for understanding the crack propagation behavior of rubber components, the methods used for the determination of tearing energies and their influencing factors are focused on by researchers in nowadays.

The aim of this work was to study the correlation of the *T*_0_ obtained from the classic Lake-Yeoh cutting method with the fatigue crack propagation measurement. A simple rubber cutting experiment was performed on an edge-notched pure shear rubber specimen under different stretch conditions. The *T*_0_ of the material was obtained based on the relationship of the cutting energy and tearing energy. In addition, under the condition of variable amplitude loading, a fatigue crack propagation experiment with the edge-notched pure shear specimen was conducted. From the crack propagation rate and the corresponding tearing energy, the *T*_0_ of the material was obtained. The *T*_0_ obtained from the cutting method was checked by the crack propagation test.

## 2. Tearing Energy and Fatigue Crack Propagation Law

The mechanical energy stored in the specimen is represented by *U*, and it will have a change (d*U*) when the crack surface area (*A*) has a unit change (d*A*). The tearing energy *T* can be defined as [14]:

(1) $$T=-\frac{dU}{dA}$$

Note that Equation (1) is calculated on the condition of a constant displacement. When a force is applied on the specimen, the tearing energy should be redefined. If a cutting implement is presented at the crack tip and then a force *f* is applied, the corresponding cutting energy *E* can be acquired [9]. Finally, the total energy *S*, which drives the crack grow, is the sum of the two terms [9]:

(2) S=T+E

where *T* is the tearing energy only caused by the displacement and *E* is caused by the cutting.

The fatigue crack propagation behavior can be divided into four regimes of increasing maximum tearing energy *T* for *R* = 0 cycles in rubber [6], where *R* is the ratio of minimum tearing energy to maximum tearing energy. In Regime I, the maximum tearing energy *T* below the incipient tearing energy *T*_0_, the crack propagation rate d*a*/d*N* is independent of the mechanical loading.

(3) $$\frac{da}{dN}=r\;\;\;\;\;\;\;\;\left(\;T\leq T_{0}\right)$$

where *a* is the crack length, *N* is the number of load cycles and *r* is the crack propagation rate. 

When *T* increases from *T*_0_ to a transition tearing energy *T*_*t*_, the relation of the rack propagation rate d*a*/d*N* and *T* satisfies the following function:

(4) $$\frac{da}{dN}=A\left(T-T_{0}\right)\;\;+r\;\;\;\;\;\;\;\left(T_{0}\leq T\leq T_{t}\right)$$

where *A* is a constant parameter of material property. 

After the transition, when the *T* increases from *T*_*t*_ to the critical tearing energy *T_c_*, the relation of the fatigue crack propagation rate with the tearing energy obeys the following power law. The *B* and *F* are constant properties of the material.

(5) $$\frac{da}{dN}=BT^{F}\;\;\;\;\;\;\left(T_{t}\leq T\leq T_{c}\right)$$

Finally, In Regime IV (*T* > *T_c_*), the crack propagation is unstable, and the crack propagation rate is essentially infinite.

(6) $$\frac{da}{dN}\to \infty \;\;\;\;\left(\;T>T_{c}\right)$$

## 3. Experiments

### 3.1. Specimen

**Figure 1:**
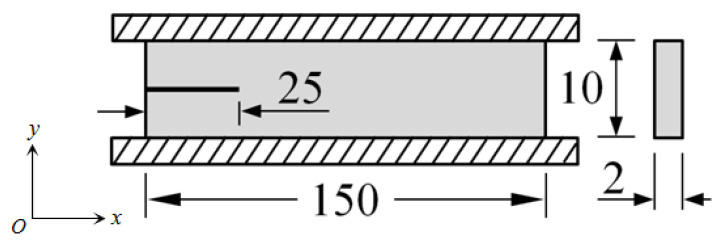
Dimensions of the test specimen (unit: mm).

The material we tested was a carbon black (CB) filled rubber with a shore-A hardness of 55, and it was generously provided by the Zhuzhou Times New Material Technology Co., Ltd., China. The main formulation of the rubber compound was as follows: 100 phr natural rubber, 30 phr CB (N774), 25 phr silica (VN3), 5 phr zinc oxide, 2 phr stearic acid, 3.5 phr antioxidant, 2 phr sulphur and 2.5 phr accelerator’promoter. The edge-notched pure shear specimen shown in Figure 1 was used for all of the tests conducted later. The specimens had widths of 150 mm, thicknesses of 2 mm and heights of 10 mm. An initial crack of length 25 mm was cut into the edge by a sharp razor blade.

### 3.2. Pure Shear Tension Experiment

**Figure 2:**
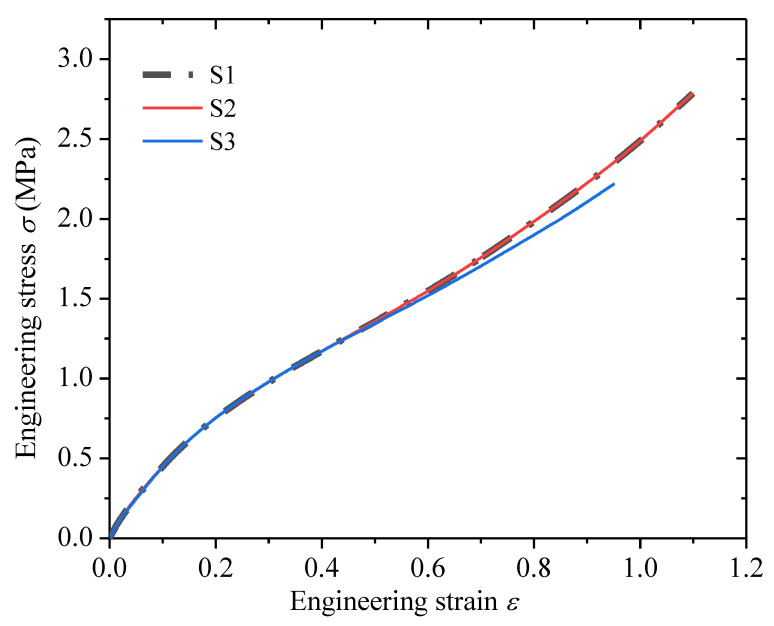
The engineering stress versus engineering strain.

The tensile mechanical behavior of a rubber material is usually studied before the rubber cutting and crack propagation experiments. The tensile behavior is used to determine the tearing energy and the maximum load imposed on the specimen during the fatigue experiment. The pure shear tension test on the edge-notched pure shear specimen shown in Figure 1 was carried out at 23 °C with an Instron machine manufactured by Instron Co. (Boston, MA, USA). The strain rate 0.01s^−1^ was used for the stretching process. In the experiment, three identical pure shear specimens (S1, S2, S3) were repeated. The measured results of engineering stress (*σ*) vs. engineering strain (*ε*) are shown in Figure 2. The strain energy density *W* can be obtained by integrating the test data through the following equation:

(7) $$W=\int_{0}^{\varepsilon }\sigma d\varepsilon$$

The tearing energy at a constant tensile strain can be calculated using the following function:

(8) T=W⋅h

where *h* is the unstrained sample height.

### 3.3. Rubber Cutting Experiment

**Figure 3:**
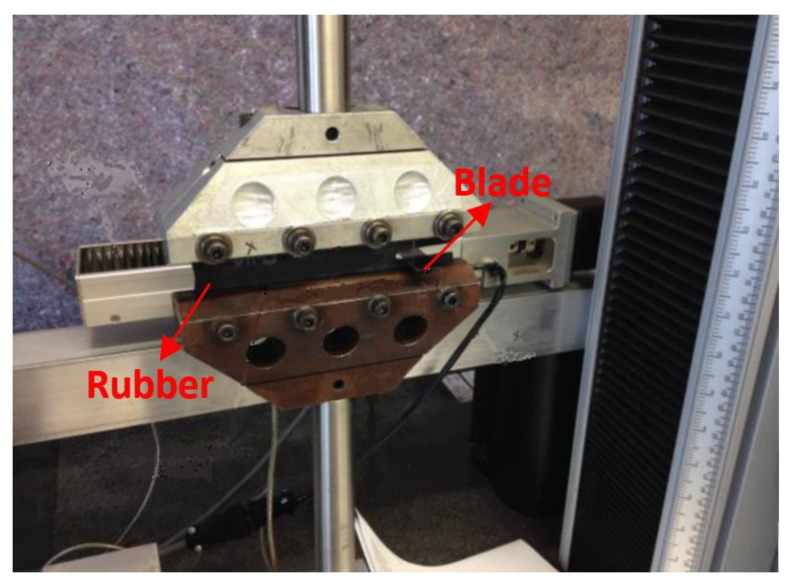
The setup of the rubber cutting experiment.

**Figure 4:**
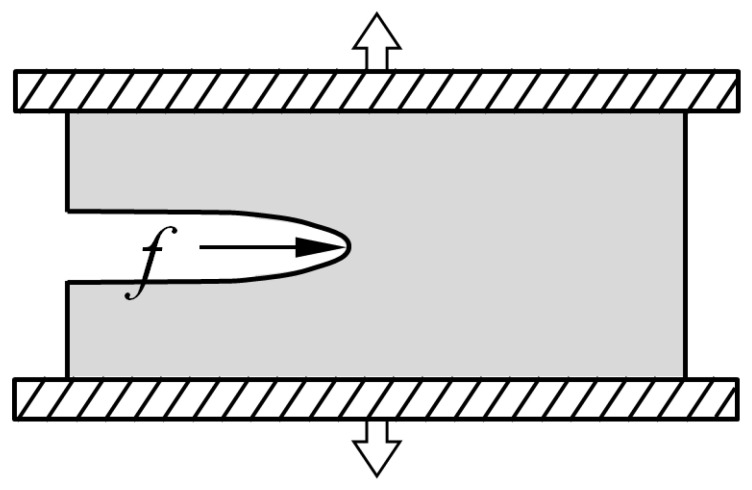
Schematic of cutting loading.

**Figure 5:**
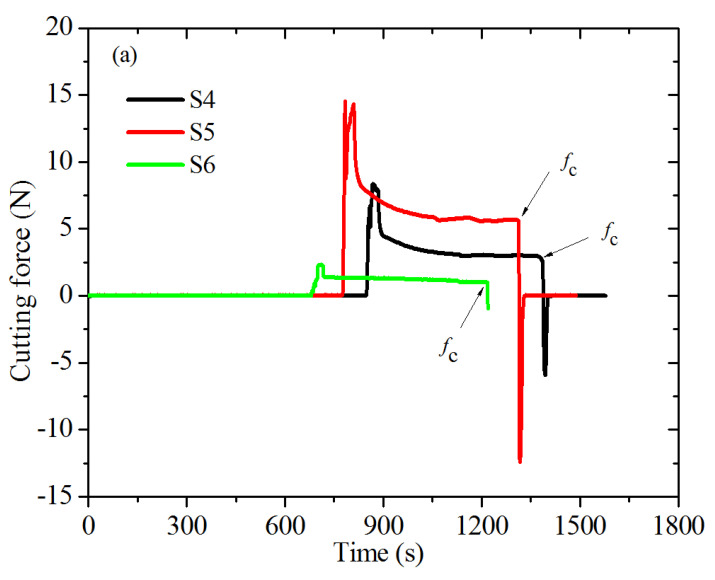

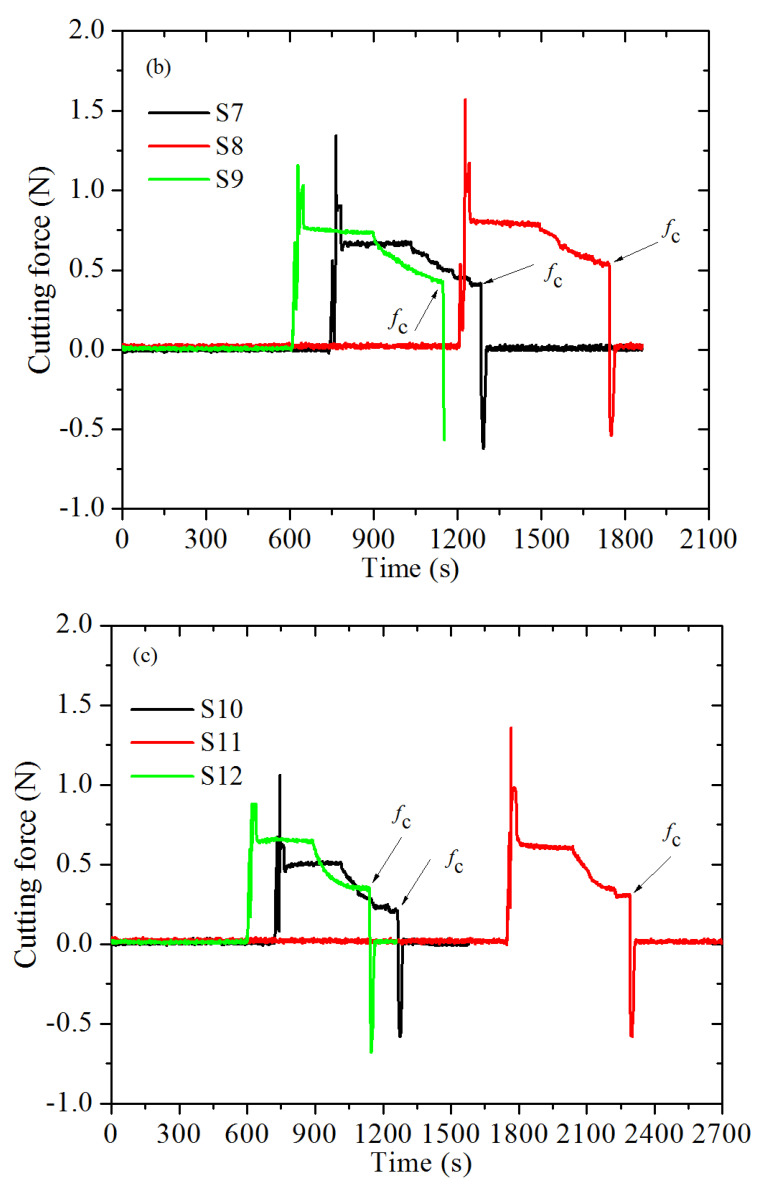
Force-time traces for various strain levels: (**a**) *ε* = 0.05, (**b**) *ε* = 0.1, (**c**) *ε* = 0.15.

Figure 3 is the setup of the rubber cutting experiment. In the rubber cutting experiment, razor blades were adopted as a cutting implement, because they can provide sharp and reasonably reproducible cutting edges. The pre-cracked planar tension test specimens were strained to three different strain levels, and the cutting force *f* was applied to the crack tip. The schematic of the loading is shown in Figure 4. At each strain level, the specimen was held at a fixed level of strain and allowed to equilibrate for a minimum of 10 min. Then, a carefully controlled, highly sharpened blade was brought into contact with the crack tip and driven to slice the specimen at a constant rate of 1 mm/min. The steady state reaction force on the blade was measured at each strain level. At each strain level, three identical pure shear specimens were used. Thus, nine specimens were needed (S4, S5, S6, S7, S8, S9, S10, S11, S12). All the cutting experiments were carried out at room temperature, 23 °C. The cutting force vs. time curves obtained during the rubber cutting experiments are shown in Figure 5.

**Figure 6:**
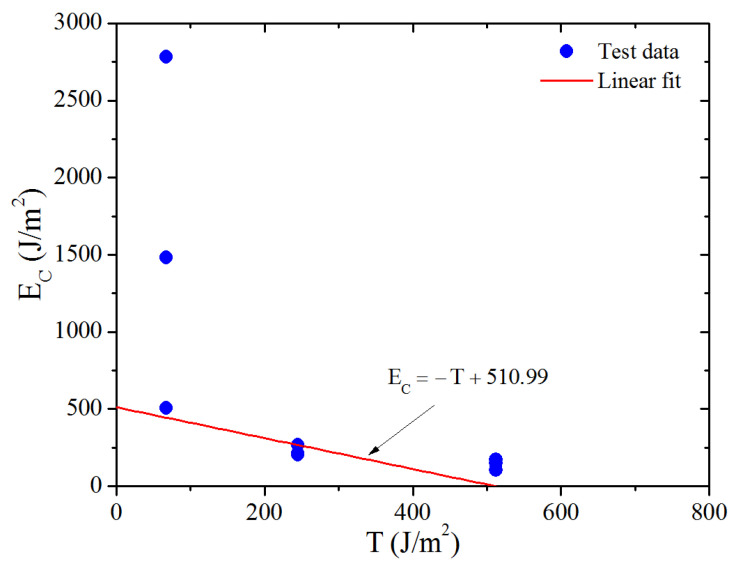
The test and linear fitting results of cutting energy versus tearing energy.

During a “catastrophic” cutting process, the cutting energy can be characterized by a critical value, *E_c_*, which can be calculated by equation [9]:

(9) $$E_{c}=f_{c}/t$$

where *f_c_* are the cutting forces before the instantaneous drop to zero during the cutting process, which are the values pointed out by the arrows in Figure 5; and *t* is the thickness of the test piece. The values of the *f_c_* and the corresponding *E_c_* calculated by Equation (9) are listed in Table 1. The result of *E_c_* varying with *T* (which can be calculated by Equations (7) and (8) based on the engineering stress versus engineering strain curve shown in Figure 2) for the three strain levels is shown in Figure 6 (solid point).

At low tearing energy *T* (<~200 J/m^2^), the relation between cutting energy and tearing energy was linear [9], and their relationship satisfies the following function:

(10) $$S_{c}=T+E_{c}$$

where *S_c_* is a constant for a given sharpness of blade and a particular vulcanizate. Moreover, values of *T*_0_ for the various vulcanizates are in direct proportion to those of *S_c_*, and their relationship is found to satisfy the equation *T*_0_ = 0.149*S_c_* from the study of Robertson (2021) [7]. Thus, to get the *T*_0_ of the material we studied, we should first obtain the *S_c_* in the low tearing energy region. Here we fitted the lowest cutting energy data points at different tear energies lay in the low tear energy range with Equation (10), as the straight line shown in Figure 6. A value of 510.99 J/m^2^ of the *S_c_* was obtained. Then, by substituting the value of *S_c_* into the equation *T*_0_ = 0.149*S_c_*, a value of 76.14 J/m^2^ for the *T*_0_ was obtained.

### 3.4. Fatigue Crack Propagation Experiment

**Figure 7:**
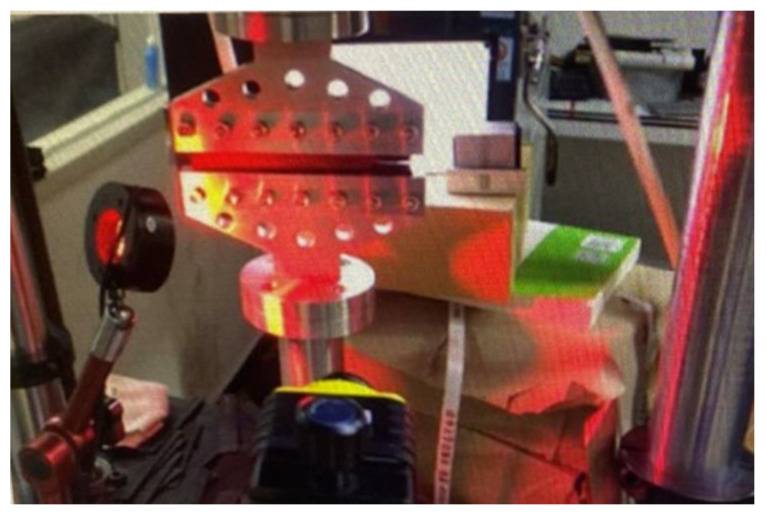
The setup of the fatigue crack propagation experiment.

**Figure 8:**
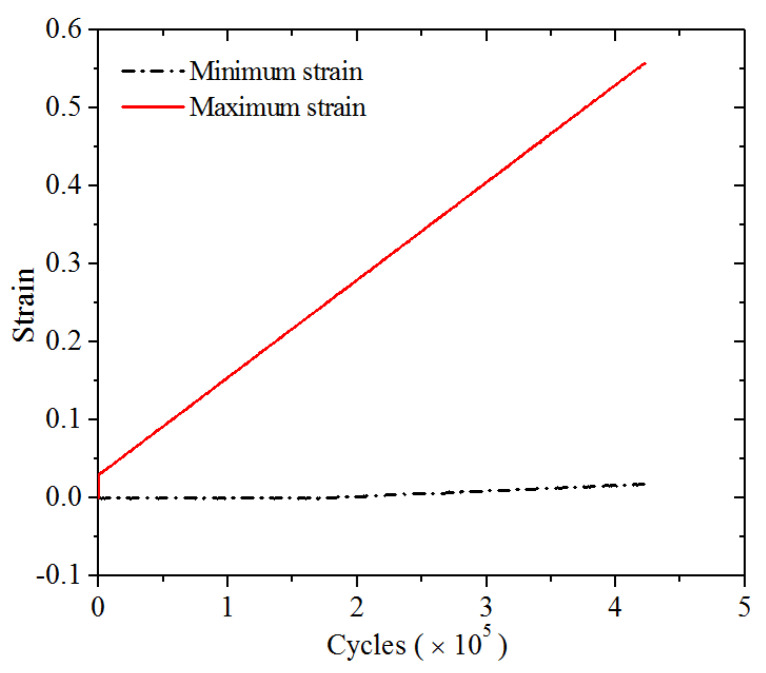
The imposed variable amplitude loading in the strain-controlled experiments.

To check the accuracy of the *T*_0_ obtained from the cutting experiment, a long-term fatigue crack propagation test was carried out. The test was conducted at room temperature (23 °C) in displacement-controlled mode. The setup of the fatigue crack propagation experiment is shown in Figure 7. The geometry of the specimens for the fatigue tests is shown in Figure 1. To quickly obtain the crack propagation test data, a variable amplitude sinusoidal load was applied to the specimen. The loading frequency was 8 Hz; *R* = 0 (*R* is defined as the ratio of minimum to maximum deformation of rubber during cycles); and the maximum strain linearly increased from 3% to 56% (see Figure 8). A digital camera was employed to measure the length of the crack. During the fatigue test, the machine was periodically stopped to record the number of cycles and measure the length of the crack.

**Figure 9:**
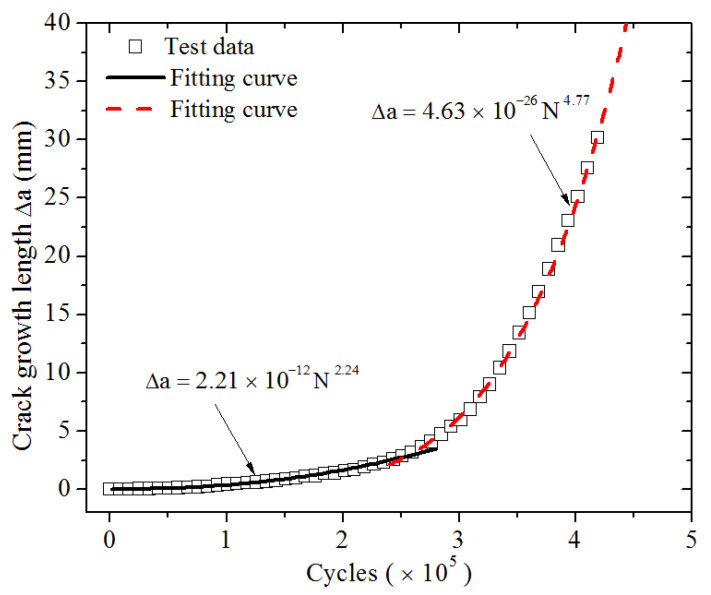
Crack contour length as a function of fatigue cycles for CB filled rubber at 23 °C.

The number of cycles *N* and the corresponding crack propagation length ∆*a* were measured, as shown in Figure 9. It can be seen that when the number of cycles was smaller than about 250,000, the crack grew slowly, whereas it grew fast when the cycles exceeded 250,000. From Figure 8, we can see that when loading was less than 250,000 cycles, the loading strain was below about 35%. The result also indicates that a critical strain of 35% exists for the material under study, above which the crack grows rapidly. This observation is in accordance with the study of Ghosh (2014) and Young (1985) [15,16].

**Figure 10:**
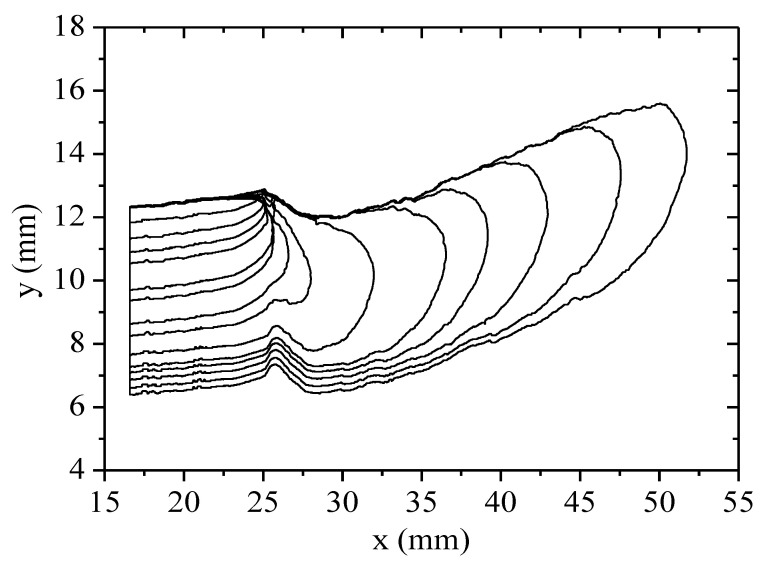
Crack front profiles for different load stages. x and y represent the coordinate positions of the points forming the crack contour, and its coordinate axis direction is shown in Figure 1.

The corresponding crack propagation path is shown in Figure 10. It shows that at the beginning of the loading cycles, the crack grew to form the natural rough crack. After a certain number of load cycles, the crack tip expanded vertically in the direction of imposed load. Thus, the crack grew slowly first, and after a certain number of load cycles, it grew fast.

The results of the crack propagation test indicate that the crack propagation has two different stages. As the loading strain increased to 35%, the crack propagation rate significantly accelerates. Thus, the relationships of the crack propagation length and the number of cycles at different strain levels below and above 35% may follow different rules. Based on the nonlinear least squares method, the relations of the crack propagation length (Δ*a*) and the number of cycles (*N*) in the two different strain regions were found to satisfy the following two power functions, respectively.

Maximum strain below 35%:

(11) $$\Delta a=2.21\times 10^{-12}N^{2.24},\;\;R^{2}=0.996$$

Maximum strain above 35%:

(12) $$△a=4.63\times 10^{-26}N^{4.77},\;\;R^{2}=0.998$$

where *R*^2^ is the correlation coefficient of the fitting of the power functions.

**Figure 11:**
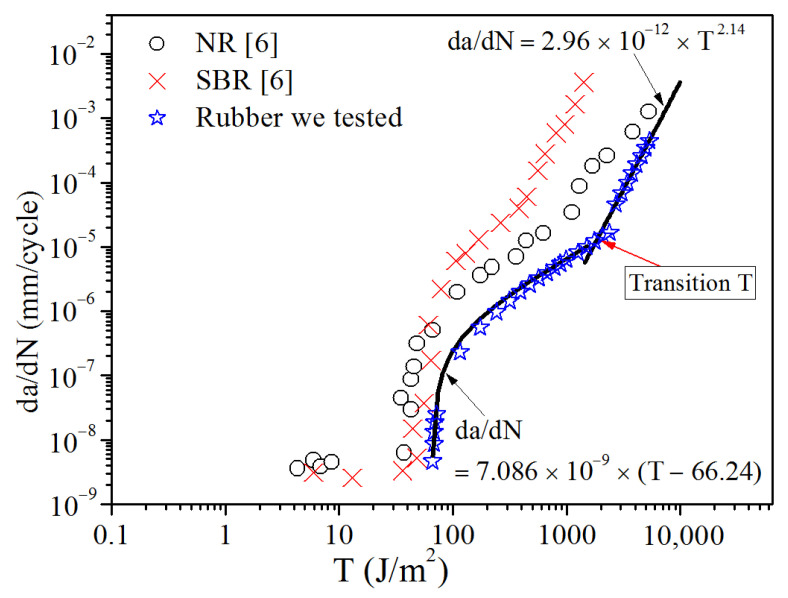
Relationship between crack propagation rate and maximum tear energy (in log-log coordinate).

The *da/dN* can be determined by the derivative of Equation (11) or Equation (12). In addition, the corresponding *T* values were obtained from Equation (8) at different crack lengths. Figure 11 represents the double logarithmic plot of tearing energy, *T* versus crack propagation rate (*da/dN*), of the studied material. We compared the test data with the typical fatigue crack propagation behavior of the unfilled SBR and NR obtained by Lake and Lindley [6], as shown in Figure 11. We can see that the d*a*/d*N* of the material we tested presents two different laws during the crack propagation, which are almost consistent with the second and third stages of the classical curves of the unfilled SBR and NR. This verifies that the method provided in this study to obtain the *da/dN* is practicable. The transition tearing value *T* is about 1800 J/m^2^. The data in the region of *T*_max_ < 1800 J/m^2^ in Figure 11 of the studied rubber were taken to determine the linear relationship of crack propagation rate and the maximum tearing energy, as shown in Equation (4). The data in the region of *T*_max_ > 1800 J/m^2^ satisfy the power relationship of crack propagation rate and maximum tearing energy, as shown in Equation (5). By fitting the corresponding test data with Equations (4) and (5), the fitting lines became those shown in Figure 11. The fitting results show that the fitting formulas (Equations (4) and (5)) are in good agreement with the measured data. A value of 66.24 J/m^2^ of *T*_0_ was obtained for the fatigue crack propagation, which is little different from the value 76.14 J/m^2^ measured by the cutting method. This indicates that the cutting method is sufficient for quantifying *T*_0_.

## 4. Conclusions

A rubber cutting experiment was conducted on an edge-notched pure shear rubber specimen. The force versus time traces at different strain levels were recorded. According to the rubber cutting test data and tearing energy theory, the incipient characteristic tearing energy *T*_0_ of the material was obtained. Under variable amplitude loading, the fatigue crack propagation experiment with edge-notched pure shear specimen was performed. A method to obtain the crack propagation rate was proposed. Additionally, from the crack propagation rate versus the corresponding tearing energy plot, the *T*_0_ of the material was determined. The values of *T*_0_ obtained from the fatigue crack propagation and cutting method are a little different. The study indicates that the cutting method is sufficient for quantifying the *T*_0_ of the rubber material, and it is less time-consuming.

## Figures and Tables

**Table 1:** Cutting force *f*_c_ and the corresponding cutting energy *E_c_*.

Strain Levels	Sample	Cutting Force *f_c_* (N)	Cutting Energy *E_c_* (J/m^2^)
0.05	S1	2.94	1.470
0.05	S2	5.61	2.805
0.05	S3	1.01	0.505
0.10	S4	0.41	0.205
0.10	S5	0.53	0.265
0.10	S6	0.43	0.215
0.15	S7	0.22	0.110
0.15	S8	0.31	0.155
0.15	S9	0.34	0.170

## Data Availability

The data presented in this study are available on request from the corresponding author.
